# Functional Characterization of Obesity-Associated Variants Involving the α and β Isoforms of Human SH2B1

**DOI:** 10.1210/en.2014-1264

**Published:** 2014-06-27

**Authors:** Laura R. Pearce, Ray Joe, Michael E. Doche, Hsiao-Wen Su, Julia M. Keogh, Elana Henning, Lawrence S. Argetsinger, Elena G. Bochukova, Joel M. Cline, Sumedha Garg, Sadia Saeed, Steven Shoelson, Stephen O'Rahilly, Inês Barroso, Liangyou Rui, I. Sadaf Farooqi, Christin Carter-Su

**Affiliations:** University of Cambridge Metabolic Research Laboratories and National Institute for Health Research Cambridge Biomedical Research Centre (L.R.P., J.M.K., E.H., E.G.B., S.G., S.Sa., S.O., I.B., I.S.F.), Wellcome Trust-Medical Research Council Institute of Metabolic Science, Addenbrooke's Hospital, Cambridge, CB2 0QQ United Kingdom; Graduate Program in Cellular and Molecular Biology (R.J., C.C.-S.), Department of Molecular and Integrative Physiology (M.E.D., H.-W.S., L.S.A., J.M.C., L.R., C.C.-S.), and Division of Metabolism, Endocrinology, and Diabetes (C.C.-S.), Department of Internal Medicine, University of Michigan Medical School, Ann Arbor, Michigan 48109–5622; Joslin Diabetes Center and Department of Medicine (S.Sh.), Harvard University, Boston, Massachusetts 02115; and Wellcome Trust Sanger Institute (I.B.), Hinxton, CB10 1SA United Kingdom

## Abstract

We have previously reported rare variants in sarcoma (Src) homology 2 (SH2) B adaptor protein 1 (*SH2B1*) in individuals with obesity, insulin resistance, and maladaptive behavior. Here, we identify 4 additional *SH2B1* variants by sequencing 500 individuals with severe early-onset obesity. SH2B1 has 4 alternatively spliced isoforms. One variant (T546A) lies within the N-terminal region common to all isoforms. As shown for past variants in this region, T546A impairs SH2B1β enhancement of nerve growth factor-induced neurite outgrowth, and the individual with the T546A variant exhibits mild developmental delay. The other 3 variants (A663V, V695M, and A723V) lie in the C-terminal tail of SH2B1α. SH2B1α variant carriers were hyperinsulinemic but did not exhibit the behavioral phenotype observed in individuals with SH2B1 variants that disrupt all isoforms. In in vitro assays, SH2B1α, like SH2B1β, enhances insulin- and leptin-induced insulin receptor substrate 2 (IRS2) phosphorylation and GH-induced cell motility. None of the variants affect SH2B1α enhancement of insulin- and leptin-induced IRS2 phosphorylation. However, T546A, A663V, and A723V all impair the ability of SH2B1α to enhance GH-induced cell motility. In contrast to SH2B1β, SH2B1α does not enhance nerve growth factor-induced neurite outgrowth. These studies suggest that genetic variants that disrupt isoforms other than SH2B1β may be functionally significant. Further studies are needed to understand the mechanism by which the individual isoforms regulate energy homeostasis and behavior.

Sarcoma (Src) homology 2 (SH2) B adaptor protein 1 (SH2B1) is a member of a family of scaffold proteins implicated in signaling downstream of a variety of receptor tyrosine kinases and cytokine receptors that bind to Janus kinases (JAKs). These include receptors for leptin, insulin, GH, IGF-I, nerve growth factor (NGF), and brain-derived neurotrophic factor (reviewed in Ref. 1). In mice, targeted deletion of *Sh2b1* results in marked leptin resistance, increased food intake, severe obesity, and insulin resistance. An intermediate obesity phenotype is seen in heterozygous null mice fed a high-fat diet (2, 3), suggesting that the obesity phenotype is dosage dependent.

Given the large number of receptor tyrosine kinases and cytokine receptor/JAK complexes that bind to SH2B1 (1), dissecting the molecular mechanisms by which SH2B1 regulates energy balance and glucose homeostasis has proved challenging. SH2B1 is alternatively spliced to yield 4 isoforms (α, β, δ, and γ) that vary in length from 671 to 756 amino acids. All isoforms share a phenylalanine zipper dimerization domain, nuclear localization sequence (NLS), nuclear export sequence, Pleckstrin homology domain, and SH2 domain but exhibit unique C termini that vary in length from 40 (SH2B1β) to 125 (SH2B1α) amino acids (Figure 1) (4). The human SH2B1 isoforms have distinct expression patterns. Although the β and γ isoforms are widely expressed, the α and δ isoforms are restricted to brain regions (5). Although very little is known about the physiological relevance of the different SH2B1 isoforms, neuron-specific restoration of the β isoform in *Sh2b1* null mice rescues the obese phenotype (6).

**Figure 1.**
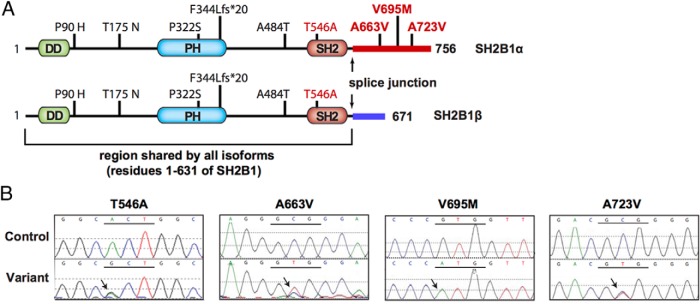
Identification of novel variants in *SH2B1*. A, Schematic representation showing the location of variants identified in *SH2B1* identified in individuals with severe obesity. The novel variants identified in this study are shown in red. Variants reported previously and the common SNP (A484T) are shown in black. DD, dimerization domain; PH, Pleckstrin homology domain; SH2, SH2 domain. B, Sequence traces of the novel variants in this study.

We previously reported rare genetic variants in *SH2B1* (P90H, T175N, P322S, and F344Lfs*20) that are located in the N-terminal 631 amino acids shared by all 4 isoforms (1–631 region). Individuals carrying these variants exhibit severe early-onset obesity and insulin resistance, and a neurobehavioral phenotype characterized by delayed speech and language development and maladaptive behavior (5). These variants disrupted SH2B1 cellular function in in vitro assays that measured GH-induced cell motility and NGF-induced neurite outgrowth. An additional SH2B1 variant (g.9483C/T), which affects only the β (T656I) and γ (P674S) isoforms, was also recently identified in obese subjects (7). This variant had no functional effect in the one assay tested (SH2B1 enhancement of leptin stimulation of signal transducer and activator of transcription 3 (STAT3) activity).

Here, we describe 4 additional *SH2B1* variants identified by sequencing a further 500 unrelated severely obese individuals from the Genetics of Obesity Study (GOOS) cohort. We performed a series of functional studies of these new variants and those previously identified by us (P90H, T175N, P322S, and F344Lfs*20) within the context of SH2B1α. There is evidence to support not only the role of rare variants in *SH2B1* in severe obesity but also of common variants with a broader role in the regulation of body mass index (BMI). As such, we also studied a common coding variant (rs7498665; A484T) that has been strongly associated with BMI in genome wide association studies (8, 9).

## Materials and Methods

### *SH2B1* variant analysis

Five hundred individuals with severe early-onset obesity (defined as a BMI SD score >3; onset, <10 y) were randomly selected from the GOOS cohort study. Primers were designed to cover the coding sequence (NM015503) and splice junctions of *SH2B1*. Variant screening was performed using PCR, followed by direct sequencing using BigDye terminator chemistry (Applied Biosystems) and analysis on an ABI 3730 automated sequencer (Applied Biosystems).

Methods for functional studies are similar to those described previously and included in the Supplemental Material (5).

## Results

### Identification of novel SH2B1 variants in severely obese individuals

We previously identified 4 variants in *SH2B1* (P90H, T175N, P322S, and F344Lfs*20) in individuals with severe early-onset obesity from the GOOS cohort (5). In the present study, we sequenced *SH2B1* in 500 additional individuals from this cohort. In addition to another individual carrying the T175N variant, we found 3 novel heterozygous variants in unrelated severely obese individuals: T546A (n = 1), A663V (n = 14), and A723V (n = 1) (Table 1). One individual was homozygous for V695M. As with the previously reported variants, the T546A variant is present in all 4 SH2B1 isoforms. However, the 3 other variants (A663V, V695M, and A723V) affect the unique C-terminal tail of SH2B1α (Figure 1). We sequenced *SH2B1* in 28 available family members of severely obese probands (Table 1). A663V variants did not cosegregate with obesity in families in a classical Mendelian manner, suggesting that *SH2B1* variants may predispose to obesity against a background of other genetic and environmental factors. There were an equal number of male and female mutation carriers (Table 1).

**Table 1.** Variants in *SH2B1* Identified in Severely Obese Individuals and the Prevalence of These Variants in the Publically Available Databases

Variant	Number of Patients	BMI				Patient BMI (SDS)	Prevalence of Variant in Publically Available Databases	
		Heterozygous Family Members (BMI)	Homozygous Family Members (BMI)	Wild-type Family Members (BMI)	Patient Neuro-behavioural Phenotype		dbSNP ID	NHLBI Exomes MAF (%)
T546A	1	29 (4.7)^a^	33^a^; 30^a^	-	28	Mild developmental delay		
(c.1636A>G)								
		Heterozygous				—	rs190981290	0.8103
A663V		34 (4.1)^a^;	38^a^; 34; 54;	50^a^; 52	32; 40;			
(c.1988C>T)		36 (3.4);	28; 27^a^; 21		27^a^;			
		22 (3.4);	52; 30; 34^a^;		23;			
		28 (3.6)^a^;	27; 32		21^a^;			
		35 (3.5);			29;			
		47 (4.0);			33^a^;			
		26 (4.3)^a^;			36;			
		43 (4.2)^a^;			26^a^;			
		26 (3.1)^a^			42;			
	14	39 (3.6);			39;	—		
		29 (5.1)^a^;			37			
		27 (4.5);						
		41 (3.7);						
		33 (6.2)						
						—		
V695M		Homo-zygous					rs375992097	0.0219
(c.2101G>A)	1	31 (5.3)	N/A	N/A	N/A	—		
A723V		Heterozygous					0	0
(c.2168C>T)	1	42	N/A	N/A	N/A	—		

*dbSNP*, SNP database dbSNP138; MAF, minor-allele frequency; NHLBI, NIH Heart, Lung, Blood Institute; exomes, http://evs.gs.washington.edu/EVS/; N/A, not available.

a Males.

Adult variant carriers were hyperinsulinaemic (mean fasting plasma insulin 128 ± 32 pmol/L; reference range, 0–60 pmol/L), but euglycaemic; liver function tests, lipid profiles, and final height were in the normal range. The individual with the T546A variant had mild developmental delay (Table 1). However, no neurobehavioral abnormalities were reported in individuals carrying the A663V, V695M, or A723V variants.

### Differences in cellular signaling mediated by human SH2B1α and β isoforms

**Figure 2.**
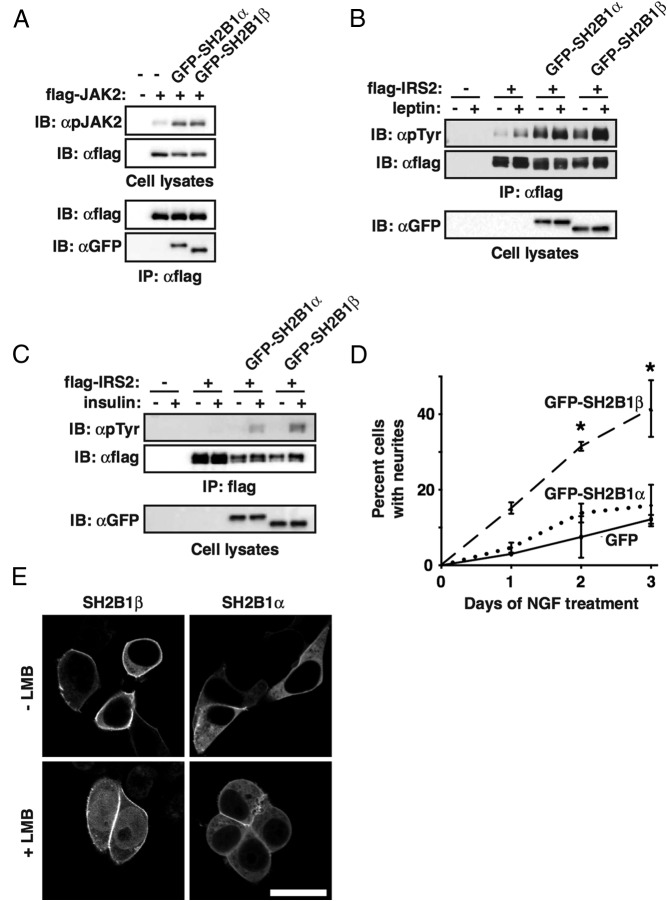
Comparison of SH2B1α and SH2B1β in vitro. A, 293 cells were transfected with the indicated constructs. Proteins in lysates were immunoprecipitated with antiflag agarose. Immunoblotting was performed using the indicated antibodies. Results are representative of 3 experiments. B, 293^LRb^ cells were transfected as shown, serum starved, and then treated with 100-ng/mL leptin for 5 minutes. Results are representative of 4 experiments. C, 293 cells were transfected with the indicated constructs, serum starved, and then stimulated with 100nM insulin for 5 minutes. Results are representative of 4 experiments. D, PC12 cells were transiently transfected with the indicated constructs and treated with 25-ng/mL NGF to induce neurite outgrowth. Results show the % of GFP+ cells with neurites greater than twice cell body length. Means ± range, n = 2 different experiments with 300 cells counted per condition per day of NGF treatment. *, *P* < .05 compared with GFP+ cells at the same time point. Statistical significance was assessed using two-way ANOVA and Bonferroni's multiple comparisons post hoc test. E, Live 293T cells transiently expressing GFP-tagged human SH2B1β or SH2B1α were incubated ± LMB (20nM) for 4 hours and imaged using fluorescent confocal microscopy. Each image is representative of 50–60 cells visualized in 2 separate experiments. Scale bar, 20 μm. IB, immunoblotting; IP, immunoprecipitation; GFP, green fluorescent protein.

We next explored the molecular mechanisms by which these variants might disrupt SH2B1 function. We first studied the ability of human SH2B1α to mediate signaling in response to a number of ligands. As a point of reference, we compared these findings with those obtained using human SH2B1β. Both SH2B1α and SH2B1β bind to JAK2 and enhance JAK2 autophosphorylation to a similar degree (Figure 2A), consistent with results of Nishi et al (10). SH2B1β is reported to bind to insulin receptor substrate (IRS) proteins and promote their tyrosyl phosphorylation in response to insulin and leptin (11, 12). Like SH2B1β, SH2B1α enhances both leptin-stimulated (Figure 2B) and insulin-stimulated (Figure 2C) tyrosyl phosphorylation of IRS2. Next, we sought to determine whether SH2B1α is involved in mediating the effects of neurotrophins such as NGF. Surprisingly, although SH2B1β enhances NGF-induced neurite outgrowth of PC12 cells (13), SH2B1α does not (Figure 2D). SH2B1β shuttles between the nucleus and the cytoplasm (14). Shuttling is thought to be necessary for SH2B1β to enhance transcription of NGF-responsive genes, such as Urokinase-type plasminogen activator receptor (uPAR), matrix metallopeptidase 3 (MMP3), and matrix metallopeptidase 10 (MMP10) (15, 16), which are implicated in neurite outgrowth of PC12 cells (16). When 293T cells expressing either SH2B1α or SH2B1β are treated with the nuclear export inhibitor, leptomycin B (LMB), only the β isoform is retained in the nucleus (Figure 2E). These results indicate that SH2B1α and SH2B1β share the ability to mediate signaling downstream of insulin, leptin, and GH. However, only the β isoform translocates to the nucleus and promotes NGF-induced neurite outgrowth.

### Functional characterization of variants affecting SH2B1α

**Figure 3.**
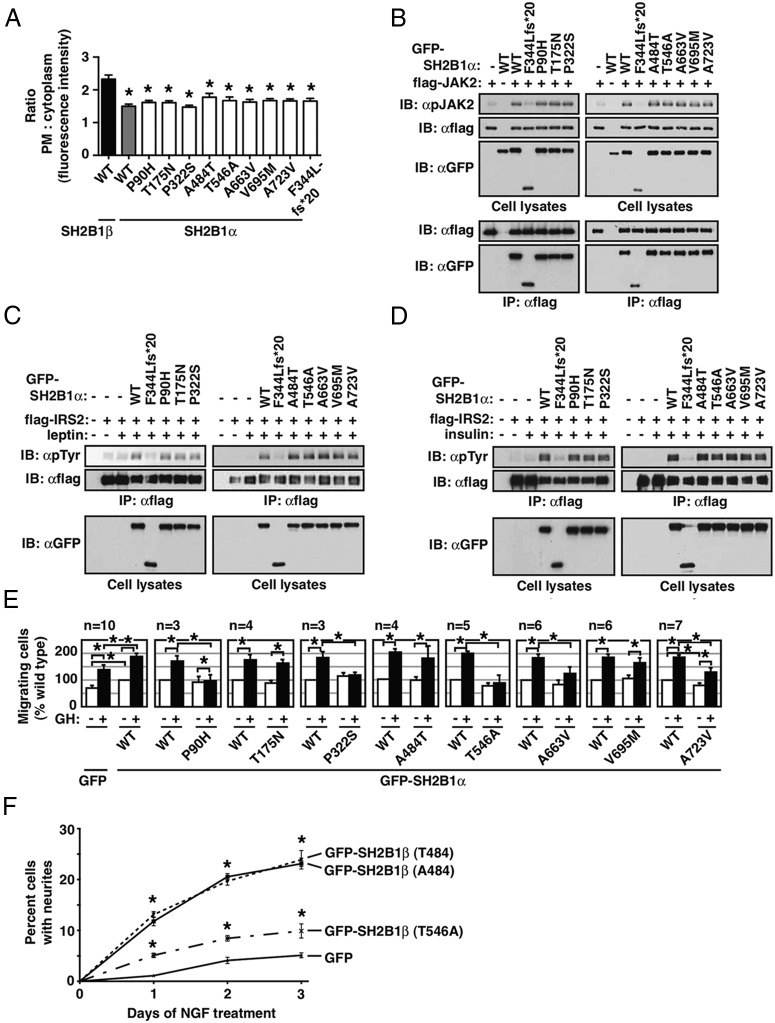
Characterization of novel human variants in SH2B1. A, Live 293T cells transiently expressing GFP-tagged rat SH2B1β, human SH2B1α WT, or human SH2B1α mutants were stained with the plasma membrane marker wheat germ agglutinin Alexa Fluor 594 and imaged by confocal microscopy. Green and red signal intensity across each cell were determined using line scan analysis (MetaVue). The ratio of the plasma membrane to cytoplasmic signal intensity is shown (mean ± SEM). *, *P* < .0001 by two-tailed, unpaired Student's *t* test compared with the ratio for rat SH2B1β WT, n = 13–19 cells/condition. B, 293 cells were transfected with the indicated constructs and resulting lysates subjected to immunoprecipitation with antiflag agarose. Immunoblotting was performed using the indicated antibodies. Results are representative of 3 experiments. C, 293^LRb^ cells were transfected as shown, serum starved, and treated with 100-ng/mL leptin for 5 minutes. Results are representative of 4 experiments. D, 293 cells were transfected with the indicated constructs, serum starved, and stimulated with 100nM insulin for 5 minutes. Results are representative of 4 experiments. E, RAW264.7 cells (2 × 10^5^ cells/well) were transiently transfected as indicated. Migration was analyzed using a transwell migration assay with or without GH (500 ng/mL) in the lower chamber for 18 hours. Values are normalized to the non-human GH-treated GFP-SH2B1α cells. Mean ± SEM from 3–10 independent experiments. *, *P* < .05 by one-tailed paired Student's *t* test. F, PC12 cells transiently expressing GFP, or GFP-tagged human SH2B1β T484, A484, or T546A, were treated with NGF (25 ng/mL) to induce neurite outgrowth. Results show the % of GFP+ cells with neurites greater than twice cell body length were counted. Means ± SEM, n = 3 different experiments with 300 cells counted per condition per day of NGF treatment. *, *P* < .05 compared with GFP cells at the same time point. GFP-SH2B1βT546A cells exhibited a statistically lower (*P* < .05) number of neurite outgrowths at days 1–3 compared with GFP-SH2B1βA484 and GFP-SH2B1βT484. Statistical significance was assessed using two-way ANOVA and Bonferroni's multiple comparisons post hoc test. IB, immunoblotting; IP, immunoprecipitation; GFP, green fluorescent protein.

We next investigated the functional consequences of variants when expressed in the SH2B1α isoform. The distribution of SH2B1α between the plasma membrane and the cytoplasm is not altered by any of the variants (Figure 3A). However, compared with SH2B1β, the intensity of SH2B1α in the plasma membrane relative to the cytoplasm is diminished (Figures 2E and 3A). Except for the frameshift mutant F344Lfs*20 that lacks the SH2 domain, none of the variants affect the ability of SH2B1α to enhance JAK2 autophosphorylation, or leptin- or insulin-induced tyrosyl phosphorylation of IRS2 (Figure 3, B–D). As reported previously for SH2B1β (5), the P90H and P332S variants reduce the ability of SH2B1α to stimulate GH-induced cell migration. The T546A, A663V, and A723V variants also reduce GH-induced cell migration (Figure 3E). T175N and V695M and the common single nucleotide polymorphism (SNP) (A484T) have no impact. Finally, we tested the effect of the variants on SH2B1 enhancement of NGF-induced neurite outgrowth. Like wild-type SH2B1α (Figure 2D), SH2B1α A663V, V695M, and A723V do not enhance NGF-induced neurite outgrowth (data not shown). However, like the previously described human variants in the 1–631 region, the T546A variant impairs the ability of SH2B1β to enhance NGF-induced neurite outgrowth. The A484T SNP has no effect on SH2B1β enhancement of NGF-induced neurite outgrowth (Figure 3F).

## Discussion

Here, we describe the identification of 4 novel variants in *SH2B1* that are present in individuals with obesity and insulin resistance. Some of the variants we found in severely obese individuals are also found in publicly available exomes (Table 1). However, because BMI and additional phenotypic information for individuals in these datasets are not available, the precise contribution of these variants to obesity remains to be established.

These findings suggest that *SH2B1* contains a spectrum of common and rare alleles that contribute to BMI and obesity predisposition with a broad range of penetrance, from low to more highly penetrant rare alleles. One variant, A663V, was identified in 14 severely obese individuals in the GOOS cohort as well as in many publically available exomes. In cells, A663V affected the ability of SH2B1 to enhance cell motility in response to GH. Therefore, it is possible that this variant may contribute to the phenotype of variant carriers. Additional genetic studies will be needed to determine whether this variant is significantly enriched in obese cohorts compared with controls. The nucleotide change that causes the A663V variant in SH2B1α also causes an amino acid change (R680C) in the δ isoform of SH2B1. Mutation of this residue in SH2B1δ impairs the ability of SH2B1δ to enhance NGF-induced neurite outgrowth (data not shown), a finding that requires further investigation.

We also studied the common coding SNP, A484T. A484T did not impact upon SH2B1α or SH2B1β in the functional assays employed here. This is consistent with a previous study (7), which was unable to demonstrate any functional consequence of the A484T SNP. It is possible that this SNP affects cellular functions of SH2B1 other than those tested or it may have subtle functional consequences that cannot be detected in the cell systems and assays we employed.

We previously showed that individuals carrying heterozygous variants in the 1–631 region of SH2B1 were hyperphagic, with a reduced final height, elevated plasma insulin levels that are disproportionate to the degree of obesity, and surprisingly, maladaptive behavior (5). Consistent with these findings, the individual carrying the T546A variant had a markedly elevated fasting plasma insulin of 123 pmol/L at age 6 years and mild developmental delay. Although individuals carrying the A663V, V695M, and A723V variants were hyperinsulinaemic, none of them were reported to display any of the behavioral characteristics reported previously. Given the small number of individuals studied, these observations need to be replicated in additional studies. We also need to determine, at the mechanistic level, whether the behavioral phenotype results from disruption of a specific SH2B1 isoform (eg, SH2B1β) or a function emanating from the 1–631 region shared by all 4 isoforms.

Three of the variants identified in this study are present within the unique C-terminal tail of SH2B1α but not SH2B1β. A limited number of studies have compared the actions of the various isoforms in vitro. All 4 SH2B1 isoforms enhanced mitogenesis and cell proliferation in response to IGF-I, insulin, and platelet-derived growth factor stimulation (4, 17). Stable expression of each isoform in NIH3T3 fibroblasts led to enhanced insulin receptor autophosphorylation and phosphorylation of IRS1 (17). In 3T3-L1 cells, all 4 isoforms enhance insulin-stimulated glucose and amino acid transport, glycogen synthesis, lipogenesis, Akt activity, and p70 S6 kinase activity (18). In all of these assays, SH2B1α was as effective as, or more effective than, SH2B1β. Thus, it was surprising to observe the inability of SH2B1α to enhance NGF-induced neurite outgrowth. The finding that the γ and δ isoforms of SH2B1 resemble SH2B1β in their ability to enhance NGF-induced neurite outgrowth (data not shown) suggests that the unique C-terminal tail of SH2B1α inhibits at least some functions mediated by the region of SH2B1 between amino acids 1–631. In contrast to its inability to promote NGF-induced neurite outgrowth, SH2B1α, like SH2B1β, was found to enhance GH-induced macrophage motility. Exactly how SH2B1 stimulates motility is not known. However, one of the proline-rich regions present in all isoforms has been shown to bind Ras-related C3 botulinum toxin substrate (19), a protein known to be involved in motility. Ligand-dependent phosphorylation of tyrosines within SH2B1 appears to be critical for SH2B1 enhancement of GH-dependent cell motility (20), suggesting that these phosphorylated tyrosines may recruit critical proteins to SH2B1 complexes. SH2B1β has also been shown to increase NGF-induced migration of PC12 cells in a wounding assay, perhaps by a protein kinase C-dependent process (21). The finding that most of the human variants impair the ability of SH2B1α and SH2B1β to enhance motility raises the possibility that regulation of the actin cytoskeleton and/or motility of cells is an important and vital component of SH2B1 function that plays a critical role in the ability of SH2B1 to regulate energy balance and the response to insulin.

SH2B1 is among a small number of adaptor proteins that undergo nucleocytoplasmic shuttling (14), although its exact role within the nucleus is not yet clear. Our previous studies suggest that neurite outgrowth requires nuclear SH2B1 (16). Human mutations, such as P90H, T175N, P322S, and F344Lfs*20, that reside in the 1–631 region of SH2B1 impair both nuclear accumulation in the presence of LMB and enhancement of NGF-induced neurite outgrowth (5). Our finding here that SH2B1α neither accumulates in the nucleus nor enhances NGF-induced neurite outgrowth is consistent with SH2B1β enhancement of neurite outgrowth requiring nuclear SH2B1β. In contrast to neurite outgrowth, SH2B1β enhancement of GH-induced macrophage motility does not require its nuclear localization (22), a finding consistent with our observation here that SH2B1α retains the ability to enhance macrophage motility despite its inability to enter the nucleus. It is possible that the unique C-terminal tail of SH2B1α interferes with the region of SH2B1 that is required for nuclear localization. Masking of the region around the NLS in SH2B1α might explain why altering amino acid 175, which lies near the NLS, impairs the ability of SH2B1β but not SH2B1α to enhance GH-induced macrophage migration.

In summary, we have identified additional SH2B1 variants in individuals with obesity and that implicate SH2B1 isoforms besides SH2B1β as important for the regulation of body weight. Further studies will be needed to understand how the distinct C-terminal tails of the α, β, γ, and δ isoforms influence SH2B1 function and their precise roles in vivo.
